# Sensor-to-Segment Calibration Methodologies for Lower-Body Kinematic Analysis with Inertial Sensors: A Systematic Review

**DOI:** 10.3390/s20113322

**Published:** 2020-06-11

**Authors:** Léonie Pacher, Christian Chatellier, Rodolphe Vauzelle, Laetitia Fradet

**Affiliations:** 1 Equipe Robotique, Biomécanique, Sport, Santé, Institut PPRIME, UPR3346 CNRS Université de Poitiers ENSMA, 86073 Poitiers, France; laetitia.fradet@univ-poitiers.fr; 2 Equipe SYstèmes et réseaux de COMmunications Optique et Radio, Institut XLIM UMR CNRS 7252, 86073 Poitiers, France; christian.chatellier@univ-poitiers.fr (C.C.); rodolphe.vauzelle@univ-poitiers.fr (R.V.)

**Keywords:** inertial measurement unit, calibration, systematic review, lower-body kinematics, human movement

## Abstract

Kinematic analysis is indispensable to understanding and characterizing human locomotion. Thanks to the development of inertial sensors based on microelectronics systems, human kinematic analysis in an ecological environment is made possible. An important issue in human kinematic analyses with inertial sensors is the necessity of defining the orientation of the inertial sensor coordinate system relative to its underlying segment coordinate system, which is referred to sensor-to-segment calibration. Over the last decade, we have seen an increase of proposals for this purpose. The aim of this review is to highlight the different proposals made for lower-body segments. Three different databases were screened: PubMed, Science Direct and IEEE Xplore. One reviewer performed the selection of the different studies and data extraction. Fifty-five studies were included. Four different types of calibration method could be identified in the articles: the manual, static, functional, and anatomical methods. The mathematical approach to obtain the segment axis and the calibration evaluation were extracted from the selected articles. Given the number of propositions and the diversity of references used to evaluate the methods, it is difficult today to form a conclusion about the most suitable. To conclude, comparative studies are required to validate calibration methods in different circumstances.

## 1. Introduction

Quantification of joint kinematics in three dimensions is essential to understanding and characterizing human body movements. It enables identification of pathological movements by comparison with asymptomatic movements [1] or the determination of performance-relevant parameters in sports [2]. For both purposes, it appears pertinent to assess kinematics in the real environment in order to characterize movement in realistic conditions [3,4]. Laboratory analyses cannot represent complete daily living behaviour, or the variety of conditions under which movement can be performed: important information can be missed [3].

Kinematics defines movements of the body segments. The human body and its movements being relatively complex, modelling is necessary at first to simplify these mechanisms. In many studies, the lower-body is modelled as linked-rigid bodies representing major bones or groups of bones in our body. The International Society of Biomechanics (ISB) has made recommendations for the creation of a lower-limb model [5,6]. This model comprises seven segments: the pelvis, the two femurs, tibia-fibula segments, and feet. The corresponding joints, namely the hips, knees, and ankles have six degrees of freedom, allowing three translations and three rotations. However, when medical imaging is not used to capture movement, translations are generally not considered [7]. To obtain the kinematics and the relative orientations between two segments, it is necessary to associate each segment with a Cartesian coordinate system. In the ISB proposal, the coordinate systems are defined by two axes obtained directly by bony landmarks. Except for the pelvis, the main axis corresponds to the longitudinal axis of the segment, whereas the secondary axis corresponds to the medio-lateral axis (often referred to as the flexion-extension axis). For the pelvis, the main axis is the medio-lateral axis, whereas the secondary axis is the longitudinal. Lastly, to define the joint kinematics by computing the three-dimensional relative orientation of the distal body-segment coordinate systems relative to the proximal orientation across a joint, Euler angle formalism is recommended, with a succession of rotations being in the Z, X’, Y’’ sequence, with the Z axis representing the proximal medio-lateral axis, the X’ the transformed antero-posterior axis, and the Y’’ axis the transformed longitudinal axis. 

The tool most used to obtain the segment movements is the optoelectronic motion-capture system [1]. This system comprises an infrared camera that follows the trajectory of markers placed on a subject’s skin. This technology has had good agreement, with an error margin inferior to 4° for the human kinematics computation on the sagittal and frontal planes [8]. However, this system is cumbersome to set up and has a relatively small measurement field. Therefore, these systems cannot be used outside laboratories to follow subjects in ecological environments [3,4] for clinical objectives, or in situ for sports application [2], as previously stated. 

The rapid development of microelectromechanical systems over the last decade has increased the popularity of inertial sensors for joint kinematics characterisation [9,10]. These developments have resulted in the miniaturization of sensors, low-power systems, and wireless technology, which represent undeniable progress. This popularity is evidenced by a large number of papers and reviews proposing to use, or attesting the use of, inertial sensors for joint kinematic analysis [9,10,11,12,13,14,15,16,17,18]. 

An inertial sensor is composed of three gyroscopes that measure angular velocity on three orthogonal axes, three accelerometers that measure linear acceleration, and usually three magnetometers that measure the intensity and direction of a magnetic field. All these measurements are expressed in a technical coordinate system associated with the inertial sensor case. A fusion of the measurements with a fusion algorithm such as a Kalman filter [19] or a complementary filter [20,21] can be used to obtain the orientation of this technical coordinate system in a global coordinate system. These inertial sensors, in the form of small boxes, are then attached to a body segment. The segment orientation is then deduced from the orientation of the sensor. For this, it is necessary to define the relative orientation of the body-segment coordinate system relative to the sensor-technical coordinate system. This procedure, referred to as the sensor-to-segment alignment or calibration, is then required to obtain joint kinematics [22]. The issue expressed here is quite different to that expressed with optoelectronic systems, since these are not positions, but linear accelerations, angular velocities, or orientations that are provided by the sensors. Therefore, methodologies proposed for optoelectronic systems cannot really be transferred to inertial sensors. 

In the current literature, different families of procedures have been proposed to obtain this relative orientation [22]. The first and easiest is manual calibration, during which the examiner is supposed to align one axis of the inertial-sensor coordinate system, the hypothesis being that this axis coincides with one sensor side: with the axis of the segment coordinate system [23]. Another approach is static calibration [24]. With this approach, the subject adopts a particular posture and the assumption is made that one of the segment axes is aligned with gravity measured by the accelerometer during this posture. In another approach, functional calibration, it is considered that the subject of the experiment is able to perform pure rotations around the segment axes being identified [25,26]. Finally, the last type of method found in the literature, referred to anatomical calibration [27,28] (sometimes called the geometric approach [28]), is close to one optoelectronic method: the CAST protocol [29,30]. In that approach, the examiner uses a device to locate anatomical landmarks or a segment axis with respect to a technical coordinate system associated with an inertial sensor placed on the device. Quite often these different methods are combined in order to define the required set of axes needed to describe the different lower-body-segment coordinate systems [31,32,33,34].

Unlike joint kinematics based on an optoelectronic system, joint kinematics based on inertial sensors do not have the significant benefit of hindsight. There is currently no consensus on which method is the most accurate or efficient in order to obtain lower-body kinematics with inertial sensors. We are still in the phase during which propositions are made without comparison or thorough reflection regarding existing propositions. In this literature review, we highlight the different methodologies proposed to analyse kinematics of human lower limbs with inertial sensors. The different calibration methods and the different postures and movements exploited will then be exposed. The mathematical methods used to obtain the segment axis will also be listed. The different evaluation methodologies proposed in order to define the robustness, the accuracy, and the repeatability of the proposals will also be presented. Finally, the discussion will propose to evoke the advantages and limits of the different methodological approaches.

## 2. Materials and Methods

In this literature review, the Preferred Reporting Items for Systematic Reviews and Meta-Analysis (PRISMA) guidelines [35] are used.

### 2.1. Inclusion and Exclusion Criteria

The aim of this review is to expose and analyse the different methodologies presented in the literature to obtain the sensor-to-segment calibration required to compute joint kinematics with the use of inertial sensors. Different inclusion criteria were selected: full papers written in English or French; studies which focus on human-movement analysis with inertial sensors and which clearly explain the sensor-to-segment calibration method. The evaluation of the proposed method included accuracy estimation by comparison with a gold standard and/or a repeatability study. Studies had to present the joint angle of the lower limb from inertial sensor measurement during an activity (walking, running, and cycling). Studies presenting only computation of spatio-temporal parameters, energy expenditure, or activity recognition were excluded.

### 2.2. Data Sources

Three databases were screened: Pubmed, Science direct, and IEEE Xplore until 28 April 2020. To collect the different studies, we used the key-word selections: (“Inertial Sensor” OR “Inertial Measurement Unit” OR “Wearable Sensor”) AND (“Lower Limb” OR “Pelvis” OR “Hip” OR “Knee” OR “Ankle”) AND (“Calibration” OR “Alignment”).

### 2.3. Study Selection

Titles and abstracts of articles selected by keywords in the databases were read by one reviewer (Léonie Pacher) to select the articles that corresponded to the inclusion criteria. After this first selection, one reviewer (Léonie Pacher) read pre-selected articles to confirm or reject the inclusion.

### 2.4. Results Synthesis

For each selected study, the type of sensor-to-segment calibration method (manual, static, anatomic, or functional), movements, or postures used, the mathematic computation used to obtain the sensor-to-segment transformation for each axis, and the accuracy validation methodology, was extracted. If done, the root mean square error and correlation coefficient between joint angles computed by the inertial sensor after sensor-to-segment calibration and a gold standard were extracted. Parameters of repeatability were also extracted for comparison. 

## 3. Results

**Figure 1:**
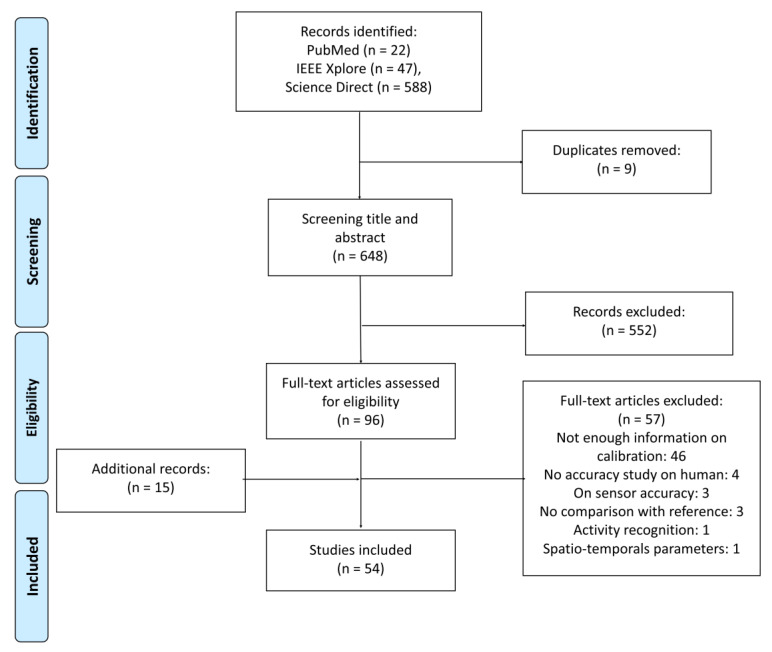
Flow diagram of search strategy.

From the database search, 646 articles were identified. After the removal of nine duplicates, the title and abstract of the remaining 638 articles were screened to select 96 articles for full-text assessment. References from included articles were screened to include relevant studies that might have been missed. After this reading, 54 articles were selected for this review. Different reasons led to the exclusion of articles: 46 did not define the calibration element, four were non-human studies, three studies examined only the accuracy of the sensors, three did not examine the accuracy of the method, and two studies dealt only with spatio-temporal parameters or physical activity recognition. Figure 1 presents a flow diagram introducing the selection procedure applied in this review.

### 3.1. Type of Sensor-to-Segment Calibration

As explained in the literature, four sensor-to-segment calibration procedures have been proposed. During manual calibration, a sensor axis is aligned with a segment axis. During static calibration, the gravity vector is considered to be aligned with one segment axis. During functional calibration, the movement rotation axis is supposed to coincide with the segment axis. Finally, anatomical calibration makes use of a device equipped with an inertial sensor to locate axis based on anatomical landmarks’ pointing.

Table 1 presents the different methods used for each possible segment axis of the lower-body segments. A preference towards the use of static methods can be observed, since 34 studies mentioned the use of a static position to define a segment axis. 

However, among these studies, 16 exclusively use the static method to obtain the different segment axes. The 18 others combine static and functional approaches to obtain all the segment axes needed to define the segment coordinate systems.

In this review, 25 studies also propose functional calibration, nine manual calibration, and five anatomical calibration.

The femur and tibia-fibula are the most studied segments, which can be explained by the fact that knee-joint kinematics are often sought after. 

If the authors had followed the ISB recommendations strictly, only the longitudinal and medio-lateral axes would have been determined. However, some proposals have also been made to define the antero-posterior segment axes. 

It must also be pointed out that in some cases methods explain how to obtain the intermediate or temporary axes required to define the desired segment axis. In other words, these temporary axes are not aligned with the segment axis, but are within an anatomical plane. We have chosen, in the following paragraphs, to explain how the segment axes were defined, even though they were obtained indirectly by the use of one or two intermediate axes. 

#### 3.1.1. Pelvis Segment Calibration

In the literature, 16 methods are proposed to determine the sensor-to-segment calibration. Regarding the pelvic longitudinal axis, during manual calibration, the authors hypothesize a perfect alignment of one inertial sensor side and the longitudinal axis of the pelvis coordinate system [36,37,38,39].

Different methods, based on static calibration, consider the pelvic longitudinal axis to be aligned with the vertical during a static upright posture [24,34,40,41,42,43,44,45]. Only one study proposes a functional calibration based on “trunk rotations”, which are unfortunately not illustrated, to determine the pelvic longitudinal axis [46]. 

An anatomical calibration of the pelvic longitudinal axis is also proposed by Picerno et al. [27]. It uses a device similar to a calliper equipped with an inertial sensor whose one axis is aligned with the longitudinal axis of the calliper. In this study, the pelvic longitudinal axis is assumed to be perpendicular to the two vectors identified with the calliper. The first vector is obtained by pointing to the right postero-superior iliac spines and the right antero-superior iliac spine with the calliper jaws; the second vector is defined by pointing to the two antero-superior iliac spines.

For the pelvic medio-lateral axis, authors using manual calibration propose to align one side of the inertial sensor case with the medio-lateral axis of the pelvis [36,37,38,39,42,47]. 

During static calibration, it is often assumed that this axis is perpendicular to two vectors lying in the sagittal plane. Three studies therefore define the medio-lateral axis as perpendicular to the gravity vector measured in two different postures: one being an upright posture and the second a sitting posture [41,48] or lying on a table [48]. 

Based on a functional approach, the medio-lateral axis is calibrated during squats [44], walking [44,49], or during trunk flexions [46]. Lebleu et al. propose original calibration movements with the “tilted to stand” and “extension stand up” options [44]. The “tilted to stand” movement starts in the sitting position with the trunk leant back and the leg extended, and consists of standing up from this position. By contrast, the “extension stand up” movement always starts in the sitting position and consists of extending the leg, bending the knees, leaning the trunk forward, and standing up. 

An anatomical calibration of the pelvic medio-lateral axis is proposed by Picerno et al. [27]. The pelvic medio-lateral axis is assumed to be parallel to the axis obtained by pointing to the two antero-superior iliac spines with the calliper jaws.

Regarding the pelvic antero-posterior axis, one study considers that it is aligned with an axis from the sensor-coordinate system during upright posture, but this is only used as a temporary vector [43].

#### 3.1.2. Femur Segment Calibration

With 36 articles, the literature on femur calibration is more substantial. For the longitudinal axis, different studies propose a manual calibration by visually aligning the femur longitudinal axis with one side of the sensor case [49,50,51,52,53]. 

Regarding the static approach, as for the pelvic longitudinal axis, the longitudinal femur axis is considered to be vertical during upright posture [31,32,33,34,40,41,43,44,45,48,54,55,56,57,58,59,60,61]. Other studies propose during this static upright posture to align the femur longitudinal axis with the predefined tibia-fibula longitudinal axis [26,36,37,38] or pelvis longitudinal axis [42].

With the Picerno et al. anatomical calibration, the femur longitudinal axis is obtained by pointing to the great trochanter and the lateral epicondyle of the knee with the calliper jaws [27]. Three other studies propose to determine this axis using the localisation on photographs of markers placed on bony landmarks [28,62,63]. For the femur medio-lateral axis, manual calibration proposes manual alignment with the sensor axis pointing laterally [47,64]. 

Static calibration methods assume here again that the axis is perpendicular to the gravity vector measured during two different static positions: the first being an upright static posture, and the second a posture during which the subject is sitting with the legs extended [41,48,59] or lying on a table [65]. During an upright static posture, the authors also align the femur medio-lateral axis with the predefined pelvis medio-lateral axis [42], or tibia-fibula medio-lateral axis [26].

For methods based on functional calibration, active or passive movements including knee flexions [36,37,38], leg flexions [43], squats [31,44,46,54,55,56], tilt to stand [44], extension stand up [44], Time Up and Go [66], walking along a straight line [33,44,49,57], cycling movements on an ergometer [34], and random circling movement around the hip and the knee joints (this last study includes kinematic constraints [67]) are used to calibrate this medio-lateral axis. 

During the Picerno et al. anatomical calibration [27], the lateral and medial epicondyle of the knee are pointed to with the calliper jaws.

A few propositions define the femur antero-posterior axis. Cordillet et al. use a static lying-down posture, during which this antero-posterior axis is considered parallel to the gravity vector [34]. 

For the methods based on the functional approach, subjects performed leg abduction/adduction [32,34,43,46,57].

#### 3.1.3. Tibia-Fibula Segment Calibration

As for femur calibration, many proposals have been made for the tibia-fibula segment, since 37 such proposals were found. For the definition of the tibia-fibula longitudinal axis with manual calibration, in some studies the examiner visually aligns this axis with one sensor-case side [52,53,61,64]. 

As for the pelvis and femur, many studies assume the longitudinal axis of the tibia-fibula to be vertical during upright posture and thus parallel to the gravity vector [31,32,33,34,36,37,38,40,41,44,45,48,54,55,57,58,59,68]. Another study proposes during this upright posture to align the tibia-fibula longitudinal axis with that obtained for the femur [42]. One study functionally defines this tibia-fibula longitudinal axis with a whole-body rotation around a vertical axis [25].

Regarding anatomical calibration, the longitudinal axis of the tibia-fibula could be defined by pointing to the lateral fibula head and the lateral malleolus with the calliper [27]. Three other studies determine the tibia-fibula longitudinal axis using localisation on photographs of markers placed on the tibia lateral condyle and the lateral malleolus [28,62,63].

The medio-lateral axis is also defined with manual calibration by considering that the axis perpendicular to the sensor case placed on the lateral side of the tibia-fibula is the segment medio-lateral axis [36,37,52,61,69]. Others, after placing the sensor in front of the tibia-fibula segment, consider that the sensor axis pointing laterally coincides with the tibia-fibula medio-lateral axis [50]. Kianifar et al. suppose that the sensor tibia-fibula axis pointing laterally is rotated 45° around the sensor longitudinal axis, the sensor being placed on the flat part of the tibia [47].

With the static calibration approach, again, the medio-lateral axis of the tibia-fibula is considered to be perpendicular to the gravity vector measured during two static postures: an upright posture as first posture and a second posture that includes lying down [34], lying on a table [41,65], or leg extended in front of the subject while sitting [41,59,65]. Another proposal for static calibration is an alignment of the medio-lateral axis of the tibia-fibula with that of the pelvis [42]. 

The functional calibrations proposed for the medio-lateral axis include active leg flexion/extension [43], active [25,34,70] or passive knee flexion [26], cycling on an ergometer [34], Time up and go exercise [66], squats [44,68], tilt to stand [44], extension stand up [44], walking [33,44,57], and random circling movement around the knee joints (this last study includes kinematic constraints [67]). Chardonnens et al. propose in their method to define the tibia-fibula medio-lateral axis as perpendicular to the acceleration at the beginning of a ski jump [55,56]. 

Regarding the Picerno et al. anatomical calibration, the tibia-fibula medio-lateral axis is obtained by pointing to the lateral and medial malleoli of the ankle [27].

A few studies also propose to determine the antero-posterior axis of the tibia-fibula. During a static calibration, which consists of a lying down posture, Cordillet et al. considers this axis to be parallel to gravity [34]. 

Functional calibrations consisting of passive rotations of the tibia-fibula in the frontal plane while the subject is seated [26], or abduction/adduction of the complete leg around the hip [31,43,57] were also described.

#### 3.1.4. Foot-Segment Calibration

Nineteen sensor-to-segment calibrations of the foot segment axes were observed. The longitudinal axis was considered to be parallel to the sensor axis pointing forward placed manually on the foot lateral side [53], whereas another study only makes this assumption to define a temporary longitudinal axis [43].

Some proposals consider that during static upright posture the longitudinal axis is aligned with the predefined antero-posterior axis of the tibia-fibula segment [36,37,42,69]. Concerning the foot medio-lateral axis, these last-cited studies propose to define this medio-lateral axis as aligned with the predefined tibia-fibula medio-lateral axis [36,37,42,69].

As for the other segments, in some studies this axis is assumed to be perpendicular to the plane represented by the gravity vector measured during two static postures: an upright posture and a sitting posture [41,65], or a lying posture [65]. 

Different functional movements are exploited to calibrate the foot medio-lateral axis: flexion/ extension of the knee [25] or ankle in sitting posture [71], tilt to stand or extension stand up movement [44], squats [44], walking [33,44], or random circling movement of the foot around the ankle joint (as previously mentioned, kinematics constraints are used in this last study [67]). 

According to the anatomical method from Picerno et al., the foot medio-lateral axis can be defined by pointing to the first and fifth metatarsal head with the calliper jaws [27].

Three studies propose to align the foot antero-posterior axis with the longitudinal axis of the tibia-fibula segment during upright posture [36,37,38], whereas many others define the foot antero-posterior axis as aligned with the gravity vector during an upright static posture [33,40,41,42,43,44,45,65]. 

A functional calibration of the foot antero-posterior axis is proposed by O’Donovan et al. with a rotation around the vertical axis of the whole body [25].

According to the anatomical method of Picerno et al., the foot antero-posterior axis is defined as being perpendicular to the plane represented by two vectors: one going from the calcaneum to the fifth metatarsal head and the second from the first to the fifth metatarsal head, both vectors being pointed to with the caliper jaws [27].

### 3.2. Mathematical Computation of Segment Axes

In the literature, different computation methods are exposed to extract the axis for sensor-to-segment calibration as presented in Table 2. For static calibration, as mentioned previously, the axis definition is based on the acceleration produced by gravity, which is measured by the accelerometers during the static posture. Generally the axis is defined as the normalised mean vector of the acceleration measured during the static acquisition [33,41,42,43,44,54,55,56,59,60,65]. 

For functional calibration, most studies define the axis based on the angular velocity measured by the sensor fixed on the segment of interest. Then the axis is considered to be the mean angular velocity vector [34,36,37,54,55,56,69] or the axis of the first principal component following a Principle Component Analysis [31,43,44,49,57,66]. Three studies use a least-square method to define an axis during functional calibration, based on the hypothesis that the angular rate perpendicular to the desired axis has to be minimal [40,67,70]. One study also applies the Principle Component Analysis to define an axis, but this time the Principle Component Analysis is applied to the measured acceleration during gait [44].

### 3.3. Evaluation of the Methods

#### 3.3.1. Method of Reference

Different systems have been used in the literature to obtain the gold standard or reference measure against which to compare the results obtained with the inertial sensors. Some studies use electro-goniometers [53,58,63], others use magnetic tracking devices (Liberty, Polhemus, Colchester, VT, USA) [26,32] or ultrasound systems (Zebris, CMS-HS, Isny, Germany) [28,62], but the majority employ optoelectronic systems [24,25,27,31,33,34,37,39,41,43,49,52,59,61,66,69].

In the vision-based motion analysis literature, there are different models proposed to obtain the joint centres and segment axes required to define the segment coordinate system. The different models used in the studies to validate the sensor calibration methodologies are shown in Table 3. It can be observed that seven studies used the Conventional Gait Model or similar [74], six used models based on clusters, namely the CAST [30] or the Starthclyde Functional Cluster [75] models, whereas the others use various functional methods [76,77,78,79,80]. The majority of these studies follow ISB recommendations to define the pelvis, hip, and ankle kinematics [6] and the Grood and Suntay recommendations for the knee kinematics [81]. None of the studies except one [66] make use of the kinematics constraint to compute the reference-joint kinematics. 

#### 3.3.2. Accuracy Assessment by Comparison with a Reference

In order to assess the quality of the proposed methods, different types of evaluations are exposed in the literature. The first approach directly compares the relative orientation between the inertial sensors and the segments-coordinate systems obtained after calibration [33,34,43]. The other proposal, and the most used, is the assessment of the difference between the resulting joint kinematics obtained with the reference and those obtained with the inertial sensors [24,26,27,28,31,32,33,34,37,41,43,50,61,62,66,69].

The characterization of the difference between the calibration matrices consists of computing an angle (helical angle) characterizing the residual transformation between the segment coordinate system obtained with the reference system and that obtained with the inertial sensors [33,34,43]. To do so, sensors and reflective markers are positioned on solid platforms affixed on the body segments. The reflective markers are then used to define a technical coordinate system, and the constant misalignment matrix between the inertial sensor coordinate system and the reference technical coordinate is evaluated. After the calibration protocol, the difference between the calibration matrices is finally evaluated, taking into account the constant misalignment matrix. Using this approach, angle errors range from 4.3° for the femur to 17° for the tibia-fibula (Table 4).

The majority of studies evaluate the accuracy of their method by comparing the joint kinematics of reference with those obtained with the inertial sensors. To evaluate this accuracy, computation of the offset existing between kinematics and comparison of the kinematic waveforms is generally performed.

Accuracy is first assessed by characterization of a global offset existing between the measured kinematics. Table 5 shows the differences in the joint kinematics based on the Root Mean Square Error (RMSE). The RMSE describes a mean offset between the joint kinematics obtained with the reference and those obtained with the inertial sensors during an activity, mainly during walking. The values vary from less than 1° up to 16°, the RMSE being generally the largest in the transverse plane (Table 5). It can be observed that some studies only compare the joint kinematics in the sagittal plane.

To quantify the offset between the reference and the inertial sensor kinematics, other studies propose to compute the difference between the mean joint angle obtained during a gait cycle with each system (Mean Absolute Error) [27,38,46,57,82] and the mean difference between the joint angles obtained with each system (Mean Absolute Variability) [37,65]. 

Differences between the range of motion are also sometimes used to quantify a difference in terms of “gain” between the methods [37,44].

The second type of evaluation used consists of qualifying waveform dissimilarity among the joint kinematic time histories. For this, the Pearson coefficient of correlation (Table 6) is the most commonly employed parameter. Globally, the studies declare higher correlation coefficients for the kinematics in the sagittal plane than in the other planes. The correlations can be considered to be good, since the lowest values reviewed are greater than 0.75. 

Two studies use a coefficient of multiple correlation (CMC) to compare the kinematic waveforms. In this case, the CMC includes comparing the kinematics for the different cycles obtained with one system (intra-system variability) and qualifying the difference between the gait cycles obtained with the other system (inter-system variability). In these studies, poorer results are found for the frontal and transverse planes for hip, knee and ankle (between 0.42 to 1 in the frontal plane and 0.45 to 0.99 in the transverse plane) than in the sagittal plane (between 0.88 to 1) [37,45,69].

It should be specified that one study proposes many different types of assessment [37]. The authors characterize not only offset and waveform differences between joint kinematics obtained with the inertial sensors and one reference, but they also compare the waveforms to other waveforms resulting from the use of different models for the reference. Finally, they also compare the kinematics of reference to the kinematics obtained by applying the proposed protocol defined for inertial sensors to data simulating inertial sensor data, but obtained via their reference system [37]. This last idea was taken up in a second study [38]. They obtain excellent kinematic similarity in the sagittal plane.

One study also applies a Bland-Altman analysis to the kinematics collected for all the subjects [66]. The authors from this study obtain a 95% confidence interval of the error on [−17°, +19°] and an average static bias offset of 0.65 (*p* < 0.001).

#### 3.3.3. Repeatability Study

Few studies presented a repeatability study. Moreover, according to the calibration method, the repeatability of the method depends on the examiner or the subject, or both. Thus, repeatability studies based on manual and anatomic sensor-to-segment calibration methods assess the examiner fixing the sensors on the segments [50], pointing the anatomic landmark with a device [27], or requiring manual intervention by the examiner for a measurement [36]. The other repeatability studies based on static or functional sensor-to-segment calibration methods assess the ability of the subjects to adopt the same static postures [32,33,65], or to perform pure rotations along the desired segment axis [26,31,32,33,43,44].

As will be evident hereafter when presenting the results, different metrics have also been proposed to assess the method repeatability where repeatability cannot be compared from one study to another. 

Manual and anatomical sensor-to-segment calibration methods are dependent on the examiner. Repeatability is therefore concentrated on intra- and inter-examiner reliability.

The standard deviation of different criteria can also be computed to assess the intra-examiner repeatability associated with a calibration method. For their manual method, Favre et al. compute the standard deviation on the range of motion of the joint angle, measured during eight walking trials performed by one subject, thus: the inertial sensors are removed and replaced between each trial by one examiner. They obtain a standard deviation of less than 2° for the knee angles [50].

For the Picerno et al. anatomical study [27], the intra- and inter-examiner repeatability is defined by the root mean square error (RMSE) computed on the angles obtained during static upright posture after six calibrations performed by the same examiner (intra-examiner repeatability) and six calibrations performed by six different examiners (inter-examiner repeatability). As usual, in this study, intra-examiner repeatability is better than inter-examiner repeatability. Maximal RMSEs are obtained for the knee internal/external rotation with 4.9° and 7.3° for intra- and inter-examiner repeatability respectively. 

For the static and functional sensor-to-segment calibration methods, the robustness of the method is dependent on the subjects. The repeatability focus is on intra-subject repeatability that determines the capacity of the subject(s) to adopt a similar posture or perform repeatable rotations during a movement. Different variables are once again used to quantify this repeatability. 

As for accuracy estimation, an initial group of assessments is based on the relative orientation between the inertial sensors and the segments-coordinate systems found following calibration, whereas a second group is based on joint kinematics. Thus, two studies proposing a method mostly based on a functional approach, characterize repeatability of the relative orientation expressed as quaternions. For this, they compute the dispersion χ corresponding to the root mean square of the angle of the residual quaternion existing between the mean quaternion and each quaternion obtained during the repeated calibration protocol. The value varies from 2.0° to 5.5° for the tibia-fibula and 2.4° to 2.9° for the femur [26,31]. Another similar proposal is based on the angle assessing the difference between the calibration matrix obtained with the inertial system and that obtained with the system of reference. The authors repeat the calibration twice and find no significant difference [33,43].

A second group of proposals is based on joint kinematics. For example, they consist of assessing differences between the mean joint angles obtained after applying each calibration result to the same trial [26,31], or assessing the joint angle differences obtained during upright posture [32]. The subjects performed the functional calibration two [26,32] or five times [31], with the mean subject difference being between 0.4° to 2.8° for hip and knee. 

Another study computes the mean absolute variability between the joint kinematics obtained following three repetitions of the protocol and the joint kinematics of reference [65]. The authors then interpret the standard error of this mean absolute variability as a representation of repeatability. They present values going from 1.8° for knee flexion to 9.7° for ankle rotation [65]. 

Lebleu et al. [44] propose to define the repeatability of different functional movements by computing the intra-class correlation coefficient and the standard error of measurement applied on the joint-movement range of motion. The subject repeated the calibration procedure three times during two different sessions. The authors obtained excellent ICC values (>0.95) on the sagittal plane, good to excellent on the transverse plane (>0.85) and good results on the frontal plane (>0.79). The standard error of measurement was smaller than 4.3°.

## 4. Discussion

Joint kinematic collection characterizing human movement seems possible nowadays in the real environment (i.e., in situ or ecologically) because of the miniaturisation of the microelectromechanical inertial sensors. The growing literature on this topic attests to the interest in these devices and their use for human-movement kinematic analysis. The present literature review focuses on different existing procedures dealing with a necessary step to obtain the lower-body joint kinematics, namely the sensor-to-segment calibration, which defines the orientation of the segment-coordinate system relative to the technical-coordinate system associated with the inertial sensor. Contrary to the use of optoelectronic motion capture systems, the use of inertial sensors to define human joint kinematics does not have the significant benefit of hindsight. Consequently, there is currently no consensus on which method is the best for lower-body kinematic analysis based on inertial sensors. As this present literature review points out, we are still in the phase during which propositions are made without comparison or thorough reflection regarding existing propositions.

Different parameters have been considered in this review: the type of sensor-to-segment calibration method, the mathematical computation of the segment axis in the sensor technical-coordinate system, and the evaluation provided by the studies.

This literature review attests to different types of sensor-to-segment calibration approach. The Table 1 show us the diversity of proposals and the difficulty to determine a standard to obtain the sensor-to-segment calibration. The different proposals have benefits and drawbacks. These elements will be discussed in the next paragraph. 

A simple example is one in which manual calibration requires the examiner to align at least one axis of the sensor-coordinate system with one axis of the segment-coordinate system. This calibration is favoured in a rehabilitation or clinical context [50,53,58,87] because of its simplicity and speed of implementation, since the subject is not required to adopt any supplementary device or specific movement or posture. However, this alignment is completely dependent on the examiner. The natural geometry of the human segments (resembling imperfect truncated cones), and the lack of visibility of the desired segment axes seem to offer the hypothetical possibility of correctly aligning the sensor axis with the segment axis. The biases of manual calibration must be considered, even more so if the subject has deformities or is an athlete with muscular prominences. The reliability and repeatability of the method is completely examiner dependent, which requires training of the examiner. There is a lack of studies on the repeatability of this method. Only one study tests the repeatability of manual calibration [50] and this only for knee kinematics. 

The second family of proposals is static calibration. This calibration requires the subject to adopt only one or two postures. This calibration can therefore also be considered as simple and rapid to set up. However, the hypothesis according to which the subject can naturally adopt a posture during which some (or all) of the lower-body segments are in an anatomically neutral position (in other words, some (or all) of the hip, knee, and ankle joint angles are null), and segment axes are aligned or perpendicular to the vertical, has not equally been discussed for the upper-body segment calibration [88]. This hypothesis is also compromised for subjects with bone deformity or muscle retraction. For such subjects, Cutti et al. [36] proposed an alternative with their Outwalk protocol. In this protocol, residual angles are measured with a goniometer, which means that an additional step and a device are required. 

The third sensor-to-segment calibration method is the functional method, which is quite similar to what is proposed in some methodologies based on optoelectronic systems [89]. It could be seen as an appropriate method to define the functional joint segment axes since the segment axis is obtained based on movements performed around the wanted axis. However, it relies on a strong hypothesis: that during the calibration movement proposed to compute the desired axis, the subjects make a pure rotation around this segment axis. Unfortunately, this hypothesis is highly questionable, as mentioned by Marin et al. for methods based on optoelectronic systems [90], and Bouvier et al. for methods based on inertial sensors, but for upper-body joint kinematics [91]. The ability to perform pure rotations along the wanted axes is especially compromised in patients with a limited range of motion or poor motor-control selectivity, as in cerebral palsy [36]. In the latter case, a solution could be to use passive functional movement performed by the examiner [26,36]. 

The fourth sensor-to-segment calibration approach is anatomical calibration. This calibration is close to some optoelectronic methods in the sense that it uses anatomical landmarks to define the segment axes. However, to use only this approach to define all the segment axes required to obtain the whole lower-body joint kinematics seems questionable. For instance, in the proposals based on this approach [27,28,62,63], the longitudinal axes of the femur and the tibia-fibula segments are not defined as a line joining the joint centres, as recommended by the ISB [6], but as a line joining external bony landmarks. One can therefore expect discrepancies between longitudinal axes defined with this anatomical method and those based on the ISB recommendations. The Picerno et al. study, however, presents excellent results in terms of joint kinematics when compared to the reference (RMSE < 4°), which can probably be explained by the fact that the medio-lateral axis defined by the anatomical landmarks is closer to the ISB recommendation [27]. With this anatomical approach, supplementary devices and trained examiners able to locate the bony landmarks are nevertheless required, which are the reasons evoked in the literature for its limited exploitation [33,34,36,43]. On the other hand, this method can be applied to all sorts of subjects-even those with muscle contracture or limited range of motion. 

Among all the reviewed studies, it can be observed that many of them [31,33,34,36,37,38,43,44,52,55,56,57,68,69,92,93,94] have combined the calibration approaches to define the required lower-body segment axes. According to their authors, these combined methods aim to retain the advantage of a simple approach, such as the static approach for some axes, while ensuring relative accuracy in axis definition with functional or anatomical methods for others. It is also mentioned that the choice of method depends on application, time, and the subject’s physical ability.

Only a few differences have been noted in terms of computation methods for obtaining the segment axis in the technical coordinate frame associated with the inertial sensor. For the functional methods, which mostly exploit angular velocity to define the axis, since no comparison has been provided, it is difficult to determine the difference between the use of the mean vector or principal component analysis. Regarding the use of angular velocity, it can be observed, which seems quite trivial, that it requires the sensor to move. Other computational possibilities could be explored, such as using a change in the sensor orientation relative to the sensor placed on the joint proximal segment, which has been proposed in the Fradet et al. study [95]. This has different advantages. Firstly, eventual parasite movements performed higher in the kinematic chain (such as pelvic or upper-leg movements when moving the lower-leg to calibrate knee flexion-extension, for example) do not influence the results. Secondly, when performing movement reversal during which angular velocity is close to zero, the axis cannot be accurately computed. This occurs twice as frequently as orientation changes. Finally, computing a segment axis does not require the segment to move. For example, it is possible to compute the rotation axis of the foot by using the orientation of the foot sensor relative to the tibia-fibula sensor during squats. Nevertheless, here again, the different possibilities should be compared. 

To validate the proposed methods, the joint kinematics obtained with inertial sensors are compared to a reference. In general, the results align closely with the kinematic profiles, especially on the sagittal plane, as attested by a Pearson correlation coefficient greater than 0.90. The offset attested by an RMSE of less than 6° is also a rather good result, since an error between 2° and 5° is considered as reasonable in methodologies based on optoelectronic systems [8]. These results are promising regarding the possibility of using inertial sensors to obtain lower-body kinematics. However, based on this literature review, it is difficult to state whether one method is better than another to perform the sensor-to-segment calibration, since no comparison of the methods has been proposed. 

As previously mentioned, joint kinematics based on inertial sensors do not have the same benefit of hindsight as joint kinematics obtained from optoelectronic systems. The methods based on optoelectronic systems have reached such a maturity that recommendations have been made to obtain hip or knee joint centres in pathological or non-pathological adults or children [96,97,98]. It can be observed that there is such a divergence between the study protocols in terms of the definition of the gold-standard and the task performed that it is impossible to reach a definitive conclusion. 

Moreover, another issue in evaluating a calibration method is the choice of the optoelectronic system as a reference. The methodologies based on optoelectronic motion capture systems are not perfect, and different elements can dramatically affect kinematic accuracy [8]. Marker misplacement can have a significant effect on kinematics [99], as does the soft tissue artefact [100]. No study has so far proposed comparing joint kinematics based on inertial sensors with joint kinematics obtained through medical imaging (the true gold standard), as has been done for methodologies based on optoelectronic motion-capture systems.

The repeatability analysis attests to the robustness of a proposed sensor-to-segment calibration method. Therefore, it is an essential step for validating a methodology. However, here again, there is no consensus on which protocols and parameters are pertinent to validate the repeatability of a method. The results provided in the literature can hardly be used to draw a conclusion about the most repeatable sensor-to-segment calibration method. The different methods must then be compared in terms of repeatability with the use of an identical protocol. 

The focus of the present review was the sensor-to-segment calibration methods. The choice of this calibration method results from a compromise between the desired kinematic accuracy for the application and the protocol, in terms of simplicity, rapidity, capacity of the subject, or the presence of a trained examiner. However, we still do not have enough objective elements to state the superiority of one approach over another, or even to provide strong recommendations. We could only establish the advantages and disadvantages of these approaches, primarily based on common sense, following the main hypothesis on which these approaches are based. New approaches might emerge. For instance, a recent study proposes the use of machine learning with a training phase based on data obtained using an optoelectronic system [101] to optimise the calibration. 

Other issues associated with the use of inertial sensors for joint kinematic computation should in future be considered in the methodology proposals. The soft tissue artefacts induced in kinematics computation by the inertial sensors are, for example, little studied [102]. The soft tissue artefact can be expected to affect to an even greater extent the kinematics obtained with inertial sensors than those obtained with optoelectronic systems, because of sensor weight. Sensor location will need to be thought through accordingly. Moreover, to limit this effect, inverse kinematics or an extended Kalman filter [103,104,105,106,107,108,109,110] can be proposed, but these different proposals should also be compared. 

## 5. Conclusions

Inertial sensors seem to be an excellent solution to analyse lower-body joint kinematics outside the laboratory (i.e., in more natural conditions). To obtain these lower-body joint kinematics, the sensor-to-segment calibration, which defines the segment coordinate system in the sensor coordinate system, is a prerequisite. Four different families of methods can be distinguished among the many proposals made in the literature to implement the sensor- to-segment calibration: the manual, static, functional, and anatomical methods. Nowadays, none of them is privileged and they are even often mixed to acquire the different lower-body segment coordinate systems. Moreover, each method has its advantages and disadvantages in terms of simplicity, speed of implementation, presupposed accuracy, or application to pathological subjects. Unfortunately, the great diversity in terms of evaluation protocols makes it impossible to compare the different methods and then to conclude about the best method. Therefore, to be able to have further discussion of the evaluation of the different methods, it will be necessary to compare the different proposals using the same protocol. 

## Figures and Tables

**Table 1:** Calibration approach used by the studies presented for each segment axis.

	Axis	Manual	Static	Functional	Anatomical
Pelvis	Long	[36,37,39,47,72]	[24,34,40,41,42,43,44,45,49]	[46]	-
	Medio-Lat	[36,37,39,42,47,72]	[24,41,42,43,44,45]	[44,46,49]	[27]
	Ant-Post	-	-	-	[27]
Femur	Long	[49,50,51,52,53]	[26,31,32,33,34,36,37,38,40,41,42,43,44,45,49,54,55,56,57,58,59,60,61,65]	-	[27,28,62,63]
	Medio-Lat	[47,50]	[26,41,42,59,65]	[26,31,33,34,36,37,38,43,44,46,49,54,55,56,57,66,67]	[27]
	Ant-Post	-	[34]	[32,34,43,46,57]	-
Tibia-fibula	Long	[50,52,53,61]	[31,32,34,36,37,38,40,41,42,43,44,45,47,54,55,56,57,58,59,60,65,73]	[25]	[27,28,62,63]
	Medio-Lat	[36,37,38,50,61]	[31,41,42,45,47,65,73]	[25,26,33,34,43,44,54,55,56,57,59,66,67,70]	[27]
	Ant-Post	-	[34]	[26,31,43,57]	-
Foot	Long	-	[36,37,38,42,45]	-	[27]
	Medio-Lat	-	[36,37,38,41,42,65]	[25,33,44,67,70]	-
	Ant-Post	[53,63]	[24,33,36,37,38,40,41,42,43,44,45,60]	[25]	[27]

**Table 2:** Computations used to obtain the segment axis.

Static Method	Functional Method			
Acceleration	Angular Velocity			Acceleration
Mean	Mean	Principle Component Analysis	Least-Squares Method	Principle Component Analysis
[33,41,42,43,44,54,55,56,59,60,65]	[34,36,37,54,55,56,69]	[31,43,44,49,57,66]	[40,67,70]	[44]

**Table 3:** Protocols and models used to obtain the segment-coordinate systems with the optoelectronic reference systems.

	Direct Kinematics		Optimized Kinematics
Conventional Gait Model or Similar	CAST Protocol or Similar	Functional Calibration	Kinematic Chain
[24,37,45,54,55,56] (Kadaba et al. [74], Plug In Gait, LAMB [83]) [47] (Motion analysis Cortex Software) [37]	[33,36,37,43,69] (CAST defined in [30]) [70] (Starthclyde functional Cluster Model defined in [75]) [37,39] (Total3D gait protocol defined in [84])	[41,82] (hip joint centre as in [76]) [85] (hip joint centre as in [77]) [61] (hip joint centre)[37] (hip, knee joint centre [86]) [31] (hip, knee, ankle joint centre as in [78]) [26,59] (knee joint centre as in [79])	[66] (Opensim)

**Table 4:** Residual angle computation characterizing the difference between the calibration matrices obtained with the calibration method and the method of reference, the Mean (standard error), in degrees, for each segment coordinate system.

	Pelvis	Femur	Tibia-Fibula	Foot
[34]		10.9 (1.6)	11.8 (2.8)	
[33]		6.1 (3.4) *	17 (4.4) *	12.2 (1.7) *
[43]	9.7 (3.44) *	4.3 (1.7) *	11 (2.6) *	9.5 (2.2) *
[43]		9.9 (3.23) *	14.7 (5.17) *	

* Read on graphs.

**Table 5:** RMSE for each study that uses it, Mean (standard error).

Reference		Pelvis			Hip			Knee			Ankle	
	Tilt	Obliquity	Rotation	Flex-Ext	Abd-Add	Int-Ext	Flex-Ext	Abd-Add	Rotation	Flex-Ext	Abd-Add	Int-Ext
[73]				8.72	4.96		6.79					
[52]				4.46	3.96		3.75					
[87]				5.8 (1.8) *^,●^			7.0 (4.0) *^,●^			5.6 (2.6) *^,●^		
[53]							5 (4.2–5.2) *^,●^			3.5 (3.9–2.5) *^,●^		
[58]							3.63(1.23)					
[41]				10.74			7.88			9.75		
[49]				2.6 (2.0)	2.7 (2.0)	6.0 (4.0)						
[61]							5.33 (2.01)					
[28]							1.30					
[62]				1.69 (0.48)			0.78 (0.17)					
[27]				0.8	1.5	1.8	1.9	2.8	3.6	1.2	2.2	3.5
[25]							0.49 (0.4) ^●^	1.6 *^,●^	3.33 (1.7) ^●^			
[26]							8.1 (5.4)	6.2 (5.1)	4.0 (4.7)			
[66]							9.69 (4.35)					
[32]							1.5 (0.4) ^◊^	1.7 (0.5) ^◊^	1.6 (0.5) ^◊^			
[38]				8.8 (4.1) ^●^	6.5 (3.5) ^●^	13.8 (8.6) ^●^	6.2 (2.0) ^●^	9.2 (6.0) ^●^	16.1 (9.8) ^●^	4.6 (3.4) ^●^	6.0 (1.1) ^●^	11.2 (2.5) ^●^
[43]				3.1 (1.2)	2.2 (0.7)	6.9 (1.4)	2.7 (0.8)	3.6 (1.0)	8 (3.3)	3.2 (1.0)	2.7 (1.1)	4.7 (2.0)
[33]							2.6 (0.8)	3.5 (1.0)	8.1 (3.5)	3.6 (1.0)	3.3 (1.4)	4.7 (1.9)
[34]							3.74 (2.99)	5.92 (2.85)	6.65 (1.94)			
[59]							3.4 (2.2)	5.6 (3.3)	5.5 (5.3)			
[44]	0.9 (0.5)	1.1 (0.9)	1.5 (1.8)	2.0 (1.2)	2.7 (2.1)	2.4 (1.5)	4.1 (3.1)	3.6 (2.3)	3.3 (2.1)	2.5 (1.7)	3.3 (2.5)	2.4 (4.3)
[2]							3.86			0.98		
[46]				3.68	2.51							
[3]				11.6 (4.8)	5.3 (1.8)	8 (3.1)	6.3 (3.2)			5.1 (2.1)	3.6 (1.3)	3.8 (1.8)

* Read on graphs; ^●^: Median (Inter Quartiles); ^◊^: Mean offset suppressed.

**Table 6:** Pearson Correlation Coefficients for each study that uses it, Mean (standard error).

Reference		Hip			Knee			Ankle	
	Flex-Ext	Abd-Add	Int-Ext	Flex-Ext	Abd-Add	Rotation	Flex-Ext	Abd-Add	Int-Ext
[73]	0.88	0.72		0.92					
[52]	0.92	0.91		0.91					
[87]	0.97			0.95			0.82		
[53]	0.99 *			0.92 *					
[58]	0.975 (0.026)								
[41]	0.98			0.97			0.78		
[65]	0.964	0.9075	0.954	0.966			0.8675	0.707	0.954
[28]				0.99					
[62]	0.99			0.99					
[27]					0.97 (0.03)				
[26]				1.00 (0.00)	0.76 (0.18)	0.85 (0.11)			
[37]	0.999	0.994	0.973	0.999			0.988	0.939	0.750
[46]	0.96	0.83							

* Read on graphs.
